# Is polycystic ovary syndrome associated with elevated muscle sympathetic nerve activity?

**DOI:** 10.1113/EP091216

**Published:** 2023-08-29

**Authors:** Alicia Duval, Will Huckins, Danielle E. Berbrier, Charlotte W. Usselman

**Affiliations:** ^1^ Department of Psychology McGill University Montreal Canada; ^2^ Cardiovascular Health and Autonomic Regulation Laboratory, Department of Kinesiology and Physical Education McGill University Montreal Canada

**Keywords:** blood pressure, cardiovascular health, muscle sympathetic nerve activity, polycystic ovary syndrome, sympathoexcitation

## Abstract

Polycystic ovary syndrome (PCOS) is a complex disorder characterized by reproductive abnormalities, cardiometabolic disturbances and a heightened risk of cardiovascular disease. A small but compelling body of research demonstrates that females with PCOS present with elevated muscle sympathetic nerve activity (MSNA) at rest. Heightened MSNA is present in lean, overweight and obese females with PCOS, but limited evidence suggests that androgens may be more strongly linked to elevated MSNA in lean females with PCOS than in obese females with PCOS. Although the specific mechanisms underlying elevated MSNA in PCOS remain elusive, sympathetic activation is implicated in the progression of several cardiovascular diseases and may contribute to the cardiovascular pathophysiology of PCOS. Encouragingly, MSNA appears responsive to non‐pharmacological intervention, making the sympathetic nervous system a promising therapeutic target to mitigate cardiovascular risk in PCOS. This brief review summarizes the existing evidence regarding elevated MSNA, cardiovascular risk profile and vascular function, as well as the potential for clinical intervention and future research directions in females with PCOS.

## Abstract (highlights)

- **What is the topic of this review?**
  The presence of elevated muscle sympathetic nerve activity in females with polycystic ovary syndrome and the implications for cardiovascular health.
- **What advance does it highlight?**
  The sympathetic nervous system likely contributes to elevated cardiovascular disease risk in females with polycystic ovary syndrome. Moreover, it presents as a promising therapeutic target for mitigating cardiovascular disease and merits further investigation.

## 1 INTRODUCTION

Polycystic ovary syndrome (PCOS) is a multifaceted endocrine disorder that affects females across the lifespan. In reproductive‐aged females, the estimated global prevalence of PCOS is between 6% and 16% (Lauritsen et al., 2014). According to the Rotterdam diagnostic criteria, the most widely accepted criteria among the medical community (Legro et al., 2013), PCOS is diagnosed according to the presence of at least two of the three following features: clinical and/or biochemical hyperandrogenism, ovarian dysfunction (i.e., oligo‐ or anovulation menstrual cycles) and polycystic ovarian morphology (Rotterdam ESHRE/ASRM‐Sponsored PCOS consensus workshop group, 2004). While there are no definitive thresholds for hyperandrogenism in PCOS, the presence of hirsutism is the predominant indicator of clinical hyperandrogenism (Rotterdam ESHRE/ASRM‐Sponsored PCOS consensus workshop group, 2004). Biochemically, free testosterone serum levels and the free T (free androgen) index are the most sensitive measurements for detecting hyperandrogenism in PCOS. Some females present with elevated dehydroepiandrosteronesulphate (DHEA‐S) and androstenedione; however, due to the scarcity of data, DHEA‐S and androstenedione are not recommended for the routine assessment of hyperandrogenism in PCOS (Rotterdam ESHRE/ASRM‐Sponsored PCOS consensus workshop group, 2004). Critical to a timely and appropriate diagnosis, females with PCOS also present with elevated cardiovascular disease (CVD) risk. Given the role of elevated sympathetic outflow as a potent (and treatable) CVD risk factor (Charkoudian & Rabbitts, 2009), it is important to understand the role of sympathetic dysfunction in PCOS. This review aims to summarize the evidence for sympathetic dysfunction that occurs in females with PCOS, explore the clinical implications of these findings, and suggest directions for future research.

## 2 CLINICAL CONTEXT: CARDIOVASCULAR MORBIDITY AND MORTALITY IN PCOS

Cardiovascular diseases are the leading cause of mortality in females, accounting for up to 35% of worldwide female deaths in 2019 (Vogel et al., 2021). A higher prevalence of traditional CVD risk factors is observed in PCOS. For example, 38–88% of females with PCOS are classified as overweight or obese (Barber & Franks, 2021). Also, the incidence of hypertension, a potent CVD risk factor, is nearly doubled (1.87‐fold) in reproductive‐aged females with PCOS (Amiri et al., 2020). Moreover, insulin resistance affects approximately 50–70% of females with PCOS, independent of body mass index (BMI; Goodarzi et al., 2011). Females with PCOS are also at an increased risk of developing endothelial dysfunction (Berbrier et al., 2023; Sprung et al., 2013), an early marker of atherosclerosis. All of these pathologies are well‐established CVD risk factors, and given their prevalence in PCOS, create a concerning profile of CVD risk in females with PCOS.

In addition to these traditional CVD risk factors, females with PCOS exhibit elevated sympathetic activity (Gui & Wang, 2017), a phenomenon that may exacerbate cardiovascular morbidity (Lansdown & Rees, 2012). This elevation in sympathetic outflow is of particular interest as it appears to interact with and is potentially amplified by other PCOS‐related comorbidities such as insulin resistance and obesity (Lansdown & Rees, 2012). Thus, elevations in sympathetic outflow may serve as an underlying physiological commonality contributing to the increased cardiovascular morbidity observed in females with PCOS.

To date, several studies have investigated the risk of cardiovascular events in females with PCOS; however, the results are mixed. A meta‐analysis by Zhang et al. (2020) revealed that PCOS was linked to an increased risk of CVD, myocardial infarction, ischemic heart disease and stroke. However, PCOS was not associated with an increased cardiovascular mortality rate, albeit the sample was relatively young with <1% identified as postmenopausal (Zhang et al., 2020). Similarly, a recent 32‐year longitudinal study reported no differences in all‐cause mortality and CVD‐related mortality in postmenopausal females with PCOS (mean age 81 years) relative to controls (Forslund et al., 2022).

Ultimately, the impact of PCOS on cardiovascular health is understudied. Most studies include only premenopausal females, in whom CVD mortality is relatively infrequent. Nevertheless, there is compelling evidence that PCOS is associated with the presence of potent CVD risk factors (Amiri et al., 2020; Barber & Franks, 2021; Berbrier et al., 2023; Goodarzi et al., 2011; Sprung et al., 2013), which likely contribute to an increased incidence of cardiovascular morbidity.

## 3 THE SYMPATHETIC NERVOUS SYSTEM IN PCOS

The microneurographic measurement of muscle sympathetic nerve activity (MSNA) is the gold standard assessment of sympathetic outflow in humans (Shoemaker et al., 2018). While there are numerous ways to quantify MSNA, the most common approach is measuring the frequency (in bursts/min) or incidence (in bursts/100 heartbeats) of bursts of integrated MSNA. Additionally, single‐unit firing rates can be examined to assess MSNA neural coding strategies (Shoemaker et al., 2018).

Elevated resting MSNA is associated with the development of CVDs such as hypertension and heart failure (Malpas, 2010). Conditions that are associated with both CVD and heightened MSNA (e.g., obesity, hypertension and elevated insulin; Kalil & Haynes, 2012) are common comorbidities in females with PCOS (Amiri et al., 2020; Barber & Franks, 2021; Goodarzi et al., 2011).

### 3.1 MSNA in obese versus lean females with PCOS

In non‐PCOS populations, elevated adiposity is associated with heightened MSNA at rest, with evidence pointing to a >50% increase in MSNA in obese versus normoweight participants (Grassi et al., 2004). In the context of PCOS, two studies have reported elevated MSNA in overweight and obese females with PCOS relative to weight‐matched controls with comparable metabolic status, both in terms of elevated MSNA burst incidence (Lambert et al., 2015) and burst frequency (Shorakae et al., 2018). The number of axons firing within a single burst of MSNA also appears to be elevated in PCOS (Lambert et al., 2015), a finding that has been linked to enhanced neurotransmitter release and vascular resistance in other populations (Shorakae et al., 2018). These studies also probed correlates of MSNA in overweight/obese PCOS and observed that the extent to which MSNA was elevated in PCOS was unrelated to markers of circulating androgens (i.e., testosterone or free androgen index; Lambert et al., 2015), age or BMI (Shorakae et al., 2018). One study has reported no differences in resting MSNA between overweight/obese females with PCOS and controls of comparable BMI (Lansdown et al., 2019), although the study was conducted in a relatively small cohort (seven females with PCOS and seven controls vs. 19 PCOS and 21 controls (Lambert et al., 2015) and 49 PCOS and 21 controls (Shorakae et al., 2018)). To the best of our knowledge, only one study to date has reported MSNA in lean females with PCOS. The study indicated that lean females with PCOS presented with elevated MSNA burst frequency and incidence relative to lean controls and that MSNA was positively associated with total and free testosterone levels (Sverrisdóttir et al., 2008). Although only two studies to date have examined the link between circulating androgens and MSNA, the findings suggest that androgens may be linked to elevated MSNA in lean (Sverrisdóttir et al., 2008) but not overweight and obese (Lambert et al., 2015) females with PCOS. Although the precise mechanisms underlying this divergence remain unclear, the excess adiposity in obese females can lead to increased aromatase activity (Hetemäki et al., 2021), thereby facilitating the conversion of androgens to oestrogen. This has the potential to attenuate the correlation between testosterone and MSNA in obese females. By contrast, in lean females with PCOS, circulating androgens are more closely associated with the severity of the syndrome, and thus with sympathetic outflow. One hypothesis suggests that the hyperandrogenic abnormality which triggers PCOS may be more severe in lean females than in their obese counterparts (Escobar‐Morreale, 2018). This hypothesis posits that obese females with PCOS may present with a less severe form of hyperandrogenism, with insulin resistance and/or obesity required as additional factors for the manifestation of the disorder.

Limited evidence suggests that PCOS may also affect MSNA responses to acute stress. Preliminary data from our laboratory indicated that lean females with PCOS demonstrated greater increases in MSNA than controls during an apnoea protocol (voluntary maximal breath hold; Usselman et al., 2019). These data are in line with research indicating that overweight and obese females with PCOS demonstrated greater MSNA burst frequency responsiveness than controls of a similar BMI during an isometric handgrip; however, we note that MSNA burst incidence, total MSNA and blood pressure responses were similar between groups (Lansdown et al., 2019). These preliminary findings suggest that females with PCOS may display increased reactivity to acute stress, as evidenced by elevated MSNA burst frequency and not by changes in MSNA burst incidence and blood pressure. However, given the small sample size of the study (*n* = 7/group), further investigation with a larger sample size is required to confirm these findings.

Given that elevations in sympathetic responsiveness to acute stress have been associated with adverse cardiovascular outcomes (Park et al., 2012), these results suggest that females with PCOS may possess an additional sympathetic‐mediated risk factor above and beyond their elevations in resting MSNA. However, to fully understand the cardiovascular risk that is associated with PCOS, it is critical to understand the extent to which elevated MSNA is coupled to vascular outcomes. To the best of our knowledge, MSNA and vascular outcomes have never been concomitantly assessed in PCOS, and thus the extent to which elevated MSNA is deleterious in women with PCOS remains unclear.

## 4 VASCULAR FUNCTION IN PCOS

The most well‐established vascular consequence of PCOS is endothelial dysfunction. Females with PCOS exhibit endothelial dysfunction regardless of BMI (Sprung et al., 2013), and the magnitude of endothelial dysfunction has been associated with circulating androgens in lean females (Berbrier et al., 2023).

Endothelial dysfunction limits vasodilatation and promotes vasoconstriction, properties that are also characteristic of high MSNA. MSNA primarily releases noradrenaline as well as smaller quantities of neuropeptide‐Y and ATP, all of which act on the vascular smooth muscle to cause vasoconstriction at the level of the arterioles (Pernow et al., 1989). This occurs when noradrenaline binds to a G protein‐coupled adrenoceptor α_1_ while ATP binds to a P2X receptor (Burnstock, 2009). Acting as a ligand‐gated ion channel, the P2X receptor enables faster transmission than the G protein‐coupled receptor (Burnstock, 2009). Neuropeptide‐Y has the ability to bind to both types of receptors. Situated on the vascular smooth muscle, these two receptor types initiate a cascade that releases calcium (Ca^2+^), causing the vascular smooth muscle to contract, leading to vasoconstriction (Burnstock, 2009). This vasoconstriction (or blunted vasodilatation) within the microvasculature can be observed at the level of the microvasculature using vascular ultrasound in the form of reduced blood flow. Notably, MSNA‐mediated vasoconstriction may not only affect vessel diameter but may also lead to increased arterial stiffness due to repeated stress on arterial walls, as evidenced in other populations (Nardone et al., 2020).

Blunted vasodilatory responses to insulin infusion have been observed in both lean/overweight (Carmassi et al., 2005) and obese females with PCOS (Paradisi et al., 2001), although another study reported no differences in blood flow responses to an oral glucose tolerance test in lean females with PCOS (Hansen et al., 2020).

Taken together, females with PCOS may have a reduced ability to vasodilate, which may occur by mechanisms related to elevated MSNA, endothelial dysfunction and/or reduced responses to insulin.

## 5 MSNA IN PCOS: RESPONSIVENESS TO INTERVENTION

Several studies have demonstrated that MSNA is responsive to intervention in overweight and obese females with PCOS. For instance, 16 weeks of combined low‐frequency electroacupuncture and regular physical exercise decreased MSNA burst frequency in females with PCOS (Stener‐Victorin et al., 2009). Likewise, MSNA burst frequency was reduced by approximately 40% in overweight and obese females with PCOS following 4–5 weeks of passive hot water immersion (Ely et al., 2019). Last, bilateral renal nerve ablation (i.e., renal denervation) reduced MSNA by 17% and 33% in two obese patients with PCOS (Schlaich et al., 2011). Together, these data indicate that interventions are effective in mitigating elevated MSNA in overweight or obese females with PCOS, although, to the best of our knowledge, these outcomes have never been assessed in lean females with PCOS.

## 6 POTENTIAL MECHANISMS CONTRIBUTING TO ELEVATED SYMPATHETIC ACTIVITY IN PCOS

The underlying mechanisms leading to increased sympathetic outflow in PCOS have yet to be fully understood, and the majority of direct evidence originates from animal studies (see Reckelhoff et al., 2022 for review). Our current understanding of elevated sympathetic activity in females with PCOS predominantly relies on indirect evidence, with potential factors including, but not limited to, central mechanisms, insulin, insulin resistance and physiological changes potentially evoked by PCOS (e.g., arterial stiffness and/or baroreflex sensitivity).

### 6.1 Central mechanisms

In humans, the modulation of MSNA is complex, involving both subcortical and cortical interactions. Subcortical regions regulating MSNA include the nucleus tractus solitarius, caudal ventrolateral medulla and rostral ventrolateral medulla, while cortical regions, including the insula, dorsolateral prefrontal cortex, posterior cingulate cortex and precuneus, are presumed to descend onto these subcortical sites to alter efferent MSNA. Although neural activity in these sites has yet to be studied in females with PCOS, current data suggest some alterations in brain structure and function. Castellano et al. (2015) demonstrated decreased regional brain glucose uptake in several regions of the frontal cortex in PCOS. Moreover, they found that females with PCOS had 10–17% lower brain volume in frontal and parietal brain regions compared to controls (Castellano et al., 2015). These data raise the question of whether central changes underlie the observed elevation in MSNA in PCOS. Investigations employing fMRI, ideally coupled with MSNA, are required in females with PCOS to fully understand this potential mechanism.

### 6.2 Insulin and insulin resistance

PCOS is often characterized by insulin resistance insofar as 75% of females with PCOS are insulin resistant, regardless of adiposity (Cassar et al., 2016). Increases in circulating insulin elevate MSNA and increase the gain of the arterial baroreflex in regulating MSNA (Young et al., 2010). Yet, in conditions such as obesity and metabolic syndrome, insulin‐mediated sympathetic activation is diminished, implying that other factors beyond insulin contribute to elevated sympathetic activity in obese individuals (Lansdown & Rees, 2012). Conversely, increased sympathetic activity may result from central insulin resistance through alterations in glucose metabolism in neurons within the hypothalamus (Landsberg, 2001). This process would subsequently lead to the suppression of the inhibitory connections between the hypothalamus and regions of the brainstem involved in sympathetic control (Landsberg, 2001; Lansdown & Rees, 2012). However, interactions between insulin and MSNA in PCOS require further investigation.

### 6.3 Arterial stiffness

PCOS has been associated with greater arterial stiffness (Trakakis et al., 2008), a condition recognized as a potent CVD risk factor (Cecelja & Chowienczyk, 2012). In other populations, positive associations between MSNA and measures of arterial stiffness have been observed (Nardone et al., 2020); however, the findings are less consistent in females (Nardone et al., 2020). Ketel et al. (2010) demonstrated that obese females, with or without PCOS, had greater arterial stiffness. In other words, this potential mechanism may be unique to overweight and obese females with PCOS and may not apply to lean females.

### 6.4 Baroreflex sensitivity

The baroreflex is an integral homeostatic mechanism that responds to beat‐to‐beat changes in blood pressure and plays a role in MSNA modulation via central mechanisms (Macefield & Henderson, 2019). In the context of PCOS, a recent study demonstrated that obese females with PCOS had lower heart rate variability (HRV; an index of suboptimal autonomic function) and lower baroreflex sensitivity than controls (Philbois et al., 2019). However, the normoweight PCOS and normoweight control group had similar baroreflex sensitivity and HRV parameters, and thus reduced baroreflex sensitivity in PCOS may be dependent on obesity. However, baroreflex sensitivity is also affected by insulin resistance, blood glucose and ovarian hormones, all of which are common to PCOS (De Melo et al., 2016; Skrapari et al., 2006; Young et al., 2010). As such, further research is required to elucidate the relationship between baroreflex sensitivity and MSNA in females with PCOS.

## 7 CAVEAT: THE SEQUENTIAL PARADOX

There is a growing body of evidence which suggests that elevated sympathetic outflow is not only a consequence of PCOS but may also actively contribute to the onset and development of the disorder. An animal study conducted by Espinoza et al. (2018) provided evidence for the role of the sympathetic nervous system in the initiation and maintenance of PCOS (Espinoza et al., 2018). In this study, pharmacological denervation of sympathetic nerves prevented the development of PCOS‐like characteristics in rats treated with oestradiol valerate, a long‐lasting oestrogen commonly used to experimentally induce PCOS in rodents. Similarly, del Campo et al. (2020) found that in vivo blockade of ovarian sympathetic nerves in rodents treated with oestradiol valerate prevented the development of PCOS‐like characteristics (del Campo et al., 2020). Moreover, suppressing ovarian sympathetic nerve activity in rodents reversed the PCOS‐like traits that were previously induced by oestradiol valerate (del Campo et al., 2020). These data suggest that elevations in the sympathetic nervous system may lead to an array of downstream effects which characterize PCOS, including increased vasoconstriction, inflammation and insulin resistance. However, the link between sympathetic outflow and the development of PCOS still warrants further investigation.

## 8 FUTURE RESEARCH DIRECTIONS

Although beyond the scope of this short review, it is important to note that PCOS is a heterogeneous disorder that encompasses multiple phenotypes (i.e., based on which diagnostic criteria are present in each patient) and which may involve different pathophysiological pathways (Escobar‐Morreale, 2018). Diagnostic criteria for PCOS remain a challenge and continue to be debated. Calls for updated diagnostic criteria suggest including body weight (Carmina, 2022) and metabolic considerations (i.e., insulin resistance; Dunaif & Fauser, 2013) to better classify different PCOS phenotypes and their consequences. The implications of these various phenotypes on MSNA, vascular outcomes and CVD risk remain poorly understood.

Despite the emerging link between PCOS and elevated MSNA, sympathetic transduction (i.e., the strength of the association between MSNA and vascular outcomes or blood pressure) has yet to be evaluated in PCOS. Elevations in MSNA concomitant with increased transduction may exacerbate cardiovascular morbidity in PCOS. That said, our preliminary data indicated that while apnoea‐induced increases in MSNA were greater in females with PCOS than controls, blood pressure responses to apnoea were similar between groups, suggesting a blunting of sympathetic transduction in PCOS (Usselman et al., 2019). Given that MSNA and/or sympathetic transduction are targets for treatments to reduce cardiovascular risk in other populations (Malpas, 2010), studies assessing these outcomes in PCOS could lead to improved evidence‐based treatments for this disorder.

To our knowledge, MSNA has not been investigated in PCOS across the lifespan. Critically, the prevalence of hypertension is higher among premenopausal females with PCOS, independent of BMI (Amiri et al., 2020). However, the risk of hypertension in PCOS declines with age, leading to the normalization of hypertension in postmenopausal females with PCOS (Amiri et al., 2020). Given the relationship between blood pressure and MSNA, MSNA may undergo distinct changes across the lifespan in PCOS. Therefore, further research is necessary to evaluate cardiovascular outcomes throughout the lifespan in females with PCOS.

## 9 CONCLUSIONS

In summary, there is good evidence that premenopausal females with PCOS exhibit elevated MSNA which may be exacerbated by elevated androgen concentrations and/or cardiometabolic disturbances. Increased MSNA may contribute to the elevated cardiovascular morbidity observed in PCOS; however, the responsiveness of MSNA to intervention makes it a promising therapeutic target to mitigate CVD risk in this population. Despite what we know about sympathetic outflow in PCOS, further research is required to understand the underlying mechanisms driving sympathoexcitation, especially considering the multifaceted and complex nature of PCOS.

## AUTHOR CONTRIBUTIONS

Alicia Duval and Charlotte W. Usselman were involved in the initial conceptualization and design of the project. All authors participated in synthesizing the data, as well as in the writing and critical revision of the manuscript. All authors have read and approved the final version of this manuscript and agree to be accountable for all aspects of the work in ensuring that questions related to the accuracy or integrity of any part of the work are appropriately investigated and resolved. All persons designated as authors qualify for authorship, and all those who qualify for authorship are listed.

## CONFLICT OF INTEREST

The authors declare no conflicts of interest.
